# Serum immunoglobulin A in pediatric appendicitis

**DOI:** 10.1038/s41598-026-39725-8

**Published:** 2026-02-13

**Authors:** Johanna Gudjonsdottir, Bodil Roth, Bodil Ohlsson, Lars Hagander, Martin Salö

**Affiliations:** 1 Department of Clinical Sciences, Pediatrics, Lund University, Lasarettsgatan 48, Lund, 221 85 Sweden; 2 Department of Surgery, Skåne University Hospital, Malmö, Sweden; 3 Department of Internal Medicine, Skåne University Hospital, Malmö, Sweden; 4 Department of Clinical Sciences, Lund University, Malmö, Sweden; 5 Department of Women’s and children’s health, Uppsala University, Uppsala, Sweden; 6 Department of Pediatric Surgery, Skåne University Hospital, Lund, Sweden

**Keywords:** Appendicitis, Serum immunoglobulin a, Pediatric, Diseases, Gastroenterology, Medical research, Risk factors

## Abstract

The pathogenesis of appendicitis is not fully understood. This study aimed to evaluate the associations between serum IgA and pediatric appendicitis. A prospective cohort study in which children ≤ 15 years with suspected appendicitis were enrolled at the Pediatric Emergency Department during 2017–2021. Blood samples were analyzed for serum IgA concentrations. Primary outcomes were appendicitis and complicated appendicitis. Associations were evaluated using univariate and multivariable logistic regression. Primary exposure was serum IgA concentrations, both in absolute concentrations as well as categorized as either normal, high or low in relation to age-dependent reference intervals. Independent variables were age, sex, symptom duration and/or presence of appendicolith. 177 children were included. Median age was 10 (IQR 8–12) years and 102 (58%) were boys. 137 (77%) had appendicitis, of which 58 (42%) were complicated. Median serum IgA among the children with appendicitis was 1.10 (IQR 0.68–1.52) g/L, compared to 1.11 (IQR 0.78–1.61) g/L in the non-appendicitis group, *p* = 0.44. Among the children with uncomplicated appendicitis, median IgA was 1.18 (0.80–1.58) g/L, compared to 0.92 (0.59–1.48) g/L among the children with complicated appendicitis, *p* = 0.07). Serum IgA was neither significantly associated with appendicitis (cOR 0.72 [95% CI 0.45–1.16], *p* = 0.18; aOR 0.70 [95% CI 0.41–1.20], *p* = 0.20) nor complicated appendicitis (cOR 0.71 [95% CI 0.41–1.21], *p* = 0.20); aOR 0.79 [95% CI 0.42–1.50], *p* = 0.47). Analyses of high and low serum IgA according to age-dependent reference intervals did not show any significant associations to appendicitis or complicated appendicitis, neither in the univariate nor the multivariable analyses. There were no significant associations between serum IgA and odds of appendicitis or complicated appendicitis in children. This does not rule out associations between appendicitis and secretory IgA found in the appendix mucosa or lumen. This should be the target for future studies on IgA and appendicitis.

## Introduction

Despite a lifetime risk of 7–9%^1^, the pathophysiological mechanisms behind appendicitis are not completely characterized. Furthermore, it is still not clear why different individuals present such vastly divergent disease courses, ranging from a mild self-limiting inflammation to an aggressive and often rapid progression towards perforation^2^.

The appendix, along with the rest of the gastrointestinal tract, is rich in lymphoid tissue, and the Gut Associated Lymphoid Tissue (GALT) provides a first line defence against intraluminal microbes and antigens^3^. The main defence mechanism of the intestines to prevent infection and inflammation is facilitated through the secretion of Immunoglobulin A (secretory IgA) from B cells in the mucosa, which helps to maintain the integrity of the mucosal barrier, prevent adhesion of pathogens to the intestinal lining by neutralizing them, promote agglutination and precipitation of microbes and pathogens, and modulate the balance between pro-inflammatory and anti-inflammatory signals^4,5^. There is a close interaction between secretory IgA and gut microbiota, generating protective immunity to various pathogens^6^. A single previous study on children has shown increased deposits of IgA in inflamed compared to healthy appendices^7^. Furthermore, interleukin 6 (IL-6) has been found to be associated with both pediatric appendicitis^8,9^and increased secretory IgA production in the appendix, at least in vitro^10^.

Although IgA is the second most abundant immunoglobulin in serum, much less is known about serum IgA compared to secretory IgA, and there does not seem to be a complete consensus regarding the functions of serum IgA^11^. Serum IgA is mostly monomeric and produced by plasma cells in bone marrow, spleen, and lymph nodes, whereas secretory IgA is mostly dimeric and produced by lamina propria^12^. However, intestinal secretory IgA may be transported across the epithelium into the blood stream^5^. Serum IgA has been characterised as an immunomodulator^13,14^as well as a secondary line of defence after communication with mucosal secretory IgA^11^. It is, however, important to underline that secretory and serum IgA are distinct entities and that serum IgA has immunological functions separate from those of secretory IgA^15^.

In this study, we investigate the associations between serum IgA, not secretory IgA, with appendicitis and complicated appendicitis in children.

## Methods

### Study design

A prospective single center cohort study with inclusion of patients aged 15 years or younger evaluated by a pediatric surgeon due to suspected appendicitis. Patient enrolment was performed between December 2017 and February 2021 at the Pediatric Emergency Department, Skåne University Hospital, Lund, Sweden – a tertiary pediatric hospital with an uptake area of 350,000 inhabitants for general surgical emergencies.

Patients were included after written informed parent/guardian consent. The study was approved by the regional ethics committee (Regionala Etikprövningsmyndigheten, Lund, Sweden, DNR 2013/614) and by the hospital review board (Skåne University Hospital, Lund, Sweden). The study was conducted in accordance with the Declaration of Helsinki.

### Inclusion and exclusion criteria

Eligible for inclusion were all children referred to the on-call pediatric surgeon due to suspected appendicitis, defined as pain in the right lower quadrant. Children with severe chronic illnesses, a history of known IgA deficiency and conditions that might influence IgA, for example major immune disorders, celiac disease, inflammatory bowel disease or chronic liver disease, were excluded. Further exclusion criteria were children who were treated with any immunomodulators, as well as with previous episodes of suspected or confirmed appendicitis.

### Data collection

The following data were collected and registered in a study protocol by the on-call pediatric surgeon assessing the patient at the Pediatric Emergency Department: current symptoms and symptom duration, findings on clinical examination, results of standard laboratory blood tests (C-reactive Protein [CRP], leukocytes and neutrophils). Data on final diagnoses, appendicitis severity, and presence of an appendicolith (noted either on preoperative ultrasonography [US] or computed tomography [CT] or intraoperatively) were then later retrieved from the patients’ medical records.

### Primary outcomes, exposure and independent variables

Primary outcomes were appendicitis and complicated appendicitis. Appendicitis diagnosis and severity were based on intraoperative findings and histopathological examination. Other diagnoses of the patients with non-appendicitis abdominal pain were based on hospital discharge notes. Uncomplicated appendicitis was defined as phlegmonous appendicitis, with infiltration of neutrophil granulocytes into the muscularis propria layer of the appendix wall on histopathological examination. Complicated appendicitis was defined as gangrenous or perforated appendicitis, and appendiceal abscess. Gangrenous appendicitis was defined as a transmural necrosis of the appendix wall, seen either intraoperatively or on histopathological examination. Perforated appendicitis was defined as the intraoperative finding of a visible hole in the appendix, or an appendicolith or free pus in the abdomen.

Primary exposure was serum IgA concentrations. Independent variables were age, sex, symptom duration (from onset of first symptoms to clinical assessment and blood sampling at the Pediatric Emergency Department), and presence of an appendicolith. Blood CRP, leukocytes, and neutrophils were captured not as potential confounders, but as indicators of the patients’ systemic inflammatory responses.

### Laboratory analyses

Blood samples were collected at the time of clinical assessment at the Pediatric Emergency Department.

Our method of blood sample management and analyses is described previously in detail^16^. Briefly, the blood samples were centrifuged, and the serum was allocated to separate containers and frozen to -80 °C and stored in a regional biobank until analysed at our research laboratory. Serum IgA was analyzed using a sandwich enzyme-linked immunosorbent assay (ELISA) kit (ab196263, Abcam, Netherlands) according to the manufacturer’s manual.

Serum IgA is presented in both absolute numbers (g/L) as well as either normal, high or low in relation to age-dependent reference intervals according to standard protocol at the Department of Clinical Chemistry, Skåne University Hospital: 21 days – 2 years: 0.01–0.50 g/L, 2–10 years: 0.4–2.5 g/L, > 10 years: 0.88–4.5 g/L^17^.

Standard inflammatory markers (CRP, leukocytes and neutrophils) were analysed according to standard protocol at the Department of Clinical Chemistry, Skåne University Hospital, Lund, Sweden^18^. Neutrophil percentages were obtained by dividing neutrophils with leukocytes.

### Statistical analyses

All statistical analyses were performed using IBM SPSS for Macintosh, version 29.0. Continuous non-normal distributed variables were reported as median with Interquartile range (IQR), with differences between two groups assessed using the Mann-Whitney U-test. Dichotomous variables were presented as frequencies and percentages, with differences between groups assessed using the Chi-squared test. Associations were assessed using logistic regression and presented as crude odds ratios (cOR) with 95% confidence intervals (CIs). Variables with statistically significant associations to the primary outcome on univariate logistic regression were included in the multivariable logistic regression models of IgA, both in absolute numbers and in relation to age-dependent reference intervals, and associations were presented as adjusted odds ratios (aOR) with 95% CIs. Standard laboratory tests were not considered potential confounders and were not included in the multivariable analyses of the associations between IgA and the primary outcomes.

## Results

**Fig. 1:**
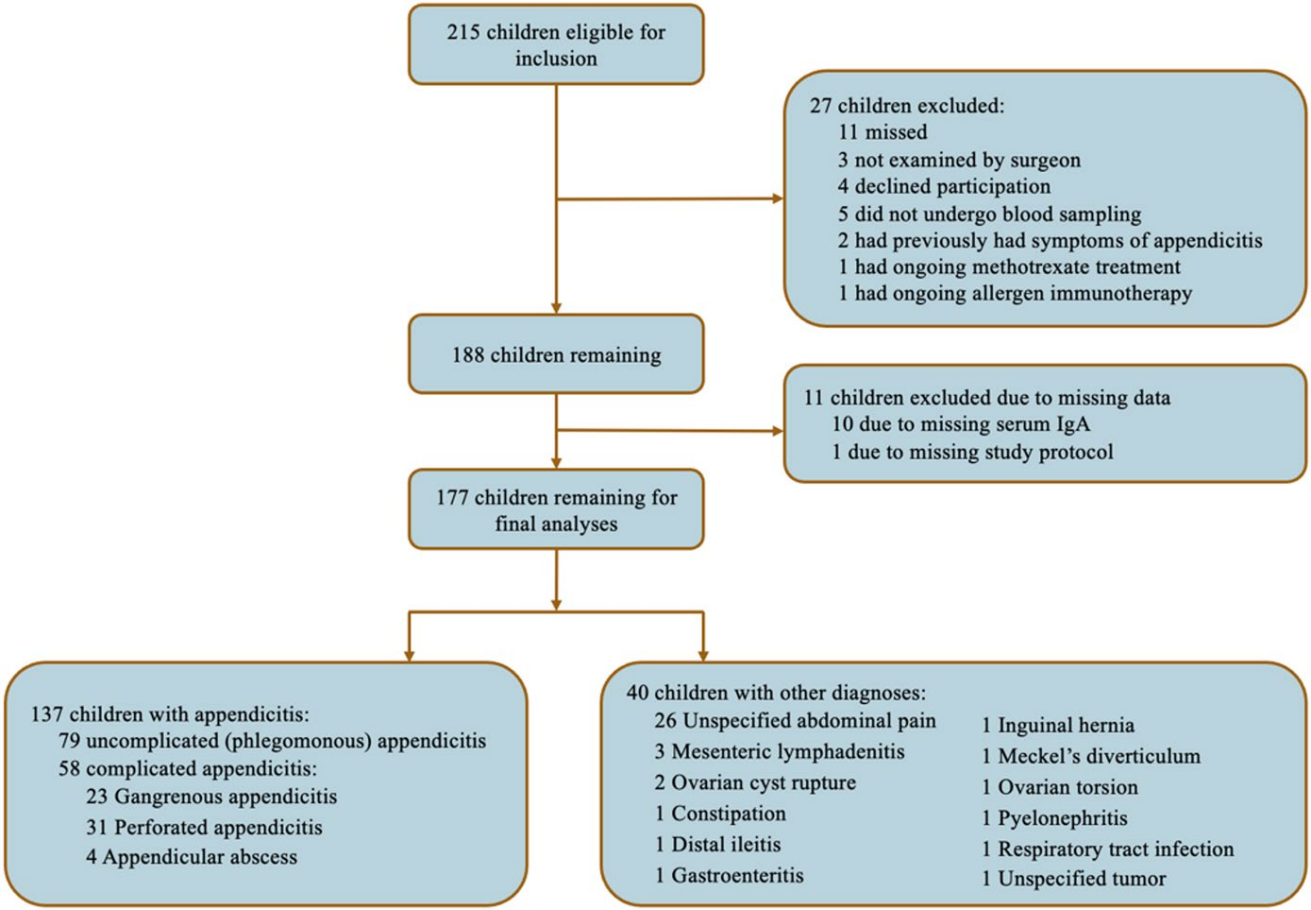
Inclusion and exclusion of children (aged < 15 years) with suspected appendicitis.

215 children were eligible for inclusion. After exclusion due to any of the exclusion criteria (*n* = 27) and missing data (*n* = 11), 177 remained for further analyses (Fig. 1). The median age of the included children was 10 (IQR 8–12) years, and 102 (58%) were male. A total of 137 children were diagnosed with appendicitis, and the diagnoses among the remaining 40 children are presented in Fig. 1. Of 137 cases of appendicitis, 79 were uncomplicated (phlegmonous) and 58 complicated (23 gangrenous, 31 perforated, and 4 abscess).

Patient demographics, symptom durations, and serum concentrations of standard inflammatory markers as well as serum IgA are presented for all included children with suspected (Table 1) and confirmed appendicitis (Table 2). Table 2 also includes appendicolith statuses, which was only investigated in the children with appendicitis. Median serum IgA among the children with appendicitis was 1.10 (IQR 0.68–1.52) g/L, compared to 1.11 (IQR 0.78–1.61) g/L in the non-appendicitis group, *p* = 0.444. Among the children with uncomplicated appendicitis, median IgA was 1.18 (0.80–1.58) g/L, compared to 0.92 (0.59–1.47) g/L among the children with complicated appendicitis, *p* = 0.072). The distribution of children with normal, high or low serum IgA did not differ significantly, neither when comparing all cases of appendicitis with the children with non-appendicitis abdominal pain (*p* = 0.802) nor cases of uncomplicated and complicated appendicitis (*p* = 0.596).

**Table 1:** Demographics, symptom durations, and serum concentrations of standard inflammatory markers and IgA of 177 children with suspected appendicitis.

	No appendicitis *n* = 40	Appendicitis *n* = 137	*p*-value
Age (years)	11 (9-12.75)	10 (8–12)	0.439
Sex (male)	16 (40%)	86 (63%)	**0.010**
Symptom duration (h)			0.082
0–24	14 (35%)	59 (43%)	
24–48	13 (33%)	50 (36%)	
48–96	9 (23%)	24 (18%)	
>96	4 (10%)	2 (1%)	
Serum IgA (g/L)	1.11 (0.78–1.61)	1.10 (0.68–1.52)	0.444
Serum IgA*			0.802
Normal	28 (70%)	96 (70%)	
High	2 (5%)	4 (3%)	
Low	10 (25%)	37 (27%)	
CRP (mg/L)	34 (16–51)	39 (20–87)	0.220
Leukocytes (x10^9^/L)	10.0 (6.7–14.8)	14.9 (12.3–18.8)	**< 0.001**
Neutrophils (x10^9^/L)	6.5 (4.2–10.6)	12.0 (9.6–15.4)	**< 0.001**
Neutrophil percentage (%)	67 (57–78)	80 (75–86)	**< 0.001**

*Serum IgA according to age dependent reference intervals: 21 days – 2 years: 0.01–0.50 g/L, 2–10 years: 0.4–2.5 g/L, > 10 years: 0.88–4.5 g/L^17^.

Values are presented as median (IQR) for continuous variables and absolute numbers (%) for grouping variables. Group differences were assessed using the Mann-Whitney U test and the Chi square test, respectively. CRP: c-reactive protein; IgA: immunoglobulin A.

*N* = 40 and 135 for symptom duration.

**Table 2:** Demographics, symptom durations, appendicolith statuses, serum concentrations of standard inflammatory markers and IgA of 137 children with confirmed appendicitis.

	Uncomplicated appendicitis *n* = 79	Complicated appendicitis *n* = 58	*p*-value
Age (years)	11 (9–13)	9 (7–12)	0.005
Sex (male)	48 (61%)	38 (66%)	0.596
Symptom duration (h)			0.063
0–24	41 (52%)	18 (31%)	
24–48	27 (34%)	23 (40%)	
48–96	10 (13%)	14 (24%)	
>96	0 (0%)	2 (3%)	
Appendicolith present	13 (18%)	21 (38%)	**0.015**
Serum IgA (g/L)	1.18 (0.80–1.58)	0.92 (0.59–1.47)	0.072
Serum IgA*			0.596
Normal	53 (67%)	43 (74%)	
High	3 (4%)	1 (2%)	
Low	23 (29%)	14 (24%)	
CRP (mg/L)	27 (17–52)	57 (32–121)	**< 0.001**
Leukocytes (x10^9^/L)	14.3 (11.7–17.8)	16.0 (13.1–19.2)	**0.046**
Neutrophils (x10^9^/L)	11.1 (9.1–14.6)	12.9 (10.3–16.0)	**0.038**
Neutrophil percentage (%)	80 (73–85)	83 (76–86)	0.051

*Serum IgA according to age dependent reference intervals: 21 days – 2 years: 0.01–0.50 g/L, 2–10 years: 0.4–2.5 g/L, > 10 years: 0.88–4.5 g/L^17^.

Values are presented as median (IQR) for continuous variables and absolute numbers (%) for grouping variables. Group differences were assessed using the Mann-Whitney U test and the Chi square test, respectively. CRP: c-reactive protein; IgA: immunoglobulin A.

*n* = 78 and 57 for symptom duration.

In the entire cohort of children with suspected appendicitis, male sex was associated with increased odds of appendicitis (cOR 2.53 [95% CI 1.23–5.20], *p* = 0.012). Symptom duration of over 96 h compared to 0–24 h was associated with a decreased odds of appendicitis (cOR 0.12 [0.02–0.71], *p* = 0.020). There were also positive associations between increased concentrations of leukocytes, neutrophils, and neutrophil percentages with increased odds of appendicitis, that remained after adjustment for sex and symptom duration. There were no significant associations with serum IgA and the odds of appendicitis, neither in the univariate nor the multivariable analyses (cOR 0.72 [0.45–1.16], *p* = 0.177, and aOR 0.70 [0.41–1.20], *p* = 0.195). High or low serum IgA in relation to age-dependent reference intervals did not associate with the odds of appendicitis (Table 3).

**Table 3:** Unadjusted and adjusted associations between independent variables and the odds for appendicitis in 177 children with suspected appendicitis.

	cOR (95% CI)	*p*-value	aOR (95% CI)	*p*-value
Age (years)	0.96 (0.85–1.08)	0.499		
Sex (male)	2.53 (1.23–5.20)	**0.012**	2.63 (1.24–5.56)^a, b,c, d,e, f^	**0.012**
Symptom duration (h)				
0–24	Ref	Ref	Ref ^a, b, c, d, e, f^	Ref
24–48	0.91 (0.39–2.12)	0.832	0.93 (0.39–2.21)	0.869
48–96	0.63 (0.24–1.66)	0.351	0.67 (0.25–1.81)	0.427
>96	0.12 (0.02–0.71)	**0.020**	0.14 (0.21–0.90)	**0.039**
Serum IgA (g/L)	0.72 (0.45–1.16)	0.177	0.70 (0.41–1.20) ^a^	0.195
Age-dependent serum IgA*				
Normal	Ref	Ref	Ref ^b^	Ref
High	0.58 (0.10–3.35)	0.546	0.45 (0.05–3.83)	0.463
Low	1.08 (0-48-2.44)	0.855	1.27 (0.53-3.00)	0.592
CRP (mg/L)	1.01 (0.99–1.01)	0.295	1.01 (0.99–1.02)^c^	0.127
Leukocytes (x10^9^/L)	1.17 (1.08–1.27)	**< 0.001**	1.17 (1.08–1.27)^d^	**< 0.001**
Neutrophils (x10^9^/L)	1.21 (1.11–1.32)	**< 0.001**	1.21 (1.10–1.33)^e^	**< 0.001**
Neutrophil percentage (%)	1.07 (1.03–1.10)	**< 0.001**	1.07 (1.03–1.10)^f^	**< 0.001**

*Serum IgA according to age-dependent reference intervals: 21 days – 2 years: 0.01–0.50 g/L, 2–10 years: 0.4–2.5 g/L, > 10 years: 0.88–4.5 g/L^17^.

^a, b,c, d,e, f^Indicates which variables were included in each multivariable logistic regression analysis.

The different inflammatory markers were included in the multivariable analysis with adjustment for sex and symptom duration one by one, and not considered potential confounders. The aOR: s for sex and symptom duration stem from the multivariable logistic regression analysis of serum IgA.

cOR: crude odds ratio; aOR: adjusted odds ratio; CRP: C-reactive protein; IgA: immunoglobulin A.

Among the children with confirmed appendicitis, increasing age was significantly associated with decreased odds of complicated appendicitis (cOR 0.84 [0.75–0.95], *p* = 0.004), while a symptom duration of 48–96 h was significantly associated with an increased risk of complicated appendicitis compared to < 24 h (cOR 3.19 [1.19–8.52], *p* = 0.021). Presence of an appendicolith increased the odds of complicated appendicitis with almost a trifold (cOR 2.82 [1.26–6.31], *p* = 0.012). Higher serum CRP, neutrophils and neutrophil percentages were also positively associated with the risk of complicated appendicitis in the univariate analyses. After adjustment for age, symptom duration and appendicolith status the association between high CRP and the odds of complicated appendicitis remained significant (aOR 1.01 [1.00-1.02], *p* = 0.027). There were no significant associations between serum IgA and the odds of complicated appendicitis (cOR 0.71 [0.41–1.21], *p* = 0.203, and aOR 0.79 [0.42–1.50], *p* = 0.473) The age-dependent stratifications of serum IgA as either high or low were not associated with the odds of complicated appendicitis, neither in the univariate or multivariable analyses (Table 4).

**Table 4:** Unadjusted and adjusted associations between independent variables and the odds for a complicated disease course in 137 children with confirmed appendicitis.

	cOR (95% CI)	*p*-value	aOR (95% CI)	*p*-value
Age (years)	0.84 (0.75–0.95)	0.004	0.85 (0.75–0.98)^a, b,c, d,e, f^	0.020
Sex (male)	1.23 (0.61–2.48)	0.569		
Symptom duration (h)				
0–24	Ref	Ref	Ref ^a, b, c, d, e, f^	Ref
24–48	1.94 (0.89–4.25)	0.098	2.29 (0.96–5.43)	0.061
48–96	3.19 (1.19–8.52)	**0.021**	2.45 (0.81–7.40)	0.111
>96	N/A	N/A	N/A	N/A
Appendicolith present	2.82 (1.26–6.31)	**0.012**	2.27 (0.94–5.49) ^a, b, c, d, e, f^	0.068
Serum IgA (g/L)	0.71 (0.41–1.21)	0.203	0.79 (0.42–1.50)^a^	0.473
Age-dependent serum IgA*				
Normal	Ref	Ref	Ref^b^	Ref
High	0.41 (0.04–4.09)	0.448	N/A	N/A
Low	0.75 (0.35–1.63)	0.468	0.65 (0.28–1.52)	0.319
CRP (mg/L)	1.02 (1.01–1.02)	**< 0.001**	1.01 (1.00-1.02)^c^	**0.027**
Leukocytes (x10^9^/L)	1.08 (0.99–1.16)	0.054	1.02 (0.94–1.11)^d^	0.600
Neutrophils (x10^9^/L)	1.08 (1.00-1.17)	**0.050**	1.03 (0.95–1.13)^e^	0.457
Neutrophil percentage (%)	1.05 (1.00-1.09)	**0.040**	1.05 (0.99–1.11)^f^	0.086

*Serum IgA according to age-dependent reference intervals: 21 days – 2 years: 0.01–0.50 g/L, 2–10 years: 0.4–2.5 g/L, > 10 years: 0.88–4.5 g/L^17^.

^a,b,c,d,e,f^ Indicates which variables were included in each multivariable logistic regression analysis.

The different inflammatory markers were included in the multivariable analysis with adjustment for age, symptom duration and appendicolith status one by one, and not considered potential confounders. The aOR: s for age and symptom duration stem from the multivariable logistic regression analysis of serum IgA.

cOR: crude odds ratio; aOR: adjusted odds ratio; CRP: C-reactive protein; IgA: immunoglobulin A.

Among the children excluded due to missing data, one child had a missing study protocol and for the other ten, the study samples never reached the laboratory, meaning they were probably never collected. To investigate any apparent bias in this step of the exclusion, the 10 children excluded due to missing IgA data were compared to the study cohort. Among the excluded children, a greater proportion were male. There were no significant differences regarding age, symptom duration, CRP, leukocytes, neutrophils, neutrophil percentage, or final diagnoses between the excluded children and the final study cohort (Supplementary Table S1).

## Discussion

To the best of our knowledge, this is the first evaluation of the associations between serum IgA and appendicitis and complicated appendicitis in children. In our cohort, serum IgA was not significantly associated to the odds of neither appendicitis nor complicated appendicitis.

The human intestine is the home of a rich variation of commensal bacteria, but also a major potential entry point for pathogens. Hence, the intestinal immune system must be able to fight a disease, while providing a favourable environment for the gut microbiota^19^– a challenging task to say the least. The appendix has a rich microbial composition that differs from the rest of the gut microbiota^20^. Secreted IgA is the most abundant antibody found on mucosal surfaces and constitutes an important part of the initial defence against pathogens, as well as the microbiota homeostatis^21^. The appendix is the main location for production of secretory IgA^20^. Serum IgA and secretory IgA are structurally and functionally different. Serum IgA is found primarily in monomeric form, while the latter is a polymer comprised of usually two, but up to as many as five, monomers^21^. Most of the human IgA is found in secreted form on mucosal surfaces, but it is also the second most abundant immunoglobulin in serum, after immunoglobulin G^15^.

There are only a few previous studies on IgA and appendicitis. One study analysed the prevalence of different immunoglobulins in appendix specimens from both rats subjected to appendicitis through lumen obstruction as well as from children with appendicitis and found increased secretory IgA deposits in the inflamed specimens compared to controls^7^. Another human study has investigated the association between serum immunoglobulins A, M and G and appendicitis, and found no association between serum IgA and appendicitis in an adult population^22^.

The present study confirmed previously known associations between for example young age^23^, and appendicolith^24^with complicated appendicitis. The commonly used biomarkers leukocytes and neutrophils were associated with the odds of appendicitis, and CRP with the odds of complicated appendicitis. This indicates systemic immune responses among the children with appendicitis and complicated appendicitis compared to those with non-appendicitis abdominal pain. Regarding serum IgA, however, there were no statistically significant associations to the odds of appendicitis or complicated appendicitis. The rationale behind the present study was primarily to investigate the pathophysiological mechanisms behind appendicitis, in terms of systemic immune responses to local inflammation. Secondarily, if serum IgA were to be associated with appendicitis and complicated appendicitis, this association could potentially serve as a clinical predictor for diagnosis and disease severity.

The lack of association between serum IgA and appendicitis and complicated appendicitis indicates a limited role of serum IgA in the inflammatory processes involved in appendicitis. Therefore, it is not useful as a standalone marker for appendicitis in children. This does not rule out associations between appendicitis and/or complicated appendicitis and altered local concentrations of secretory IgA in the appendix, since serum IgA should not be considered a surrogate marker for local mucosal responses, in accordance with the known physiology of IgA production and secretion^5,12^.

Since secretory IgA is crucial for the maintenance of mucosal integrity and prevention of infectious and inflammatory conditions in the gut^5^, the result of this study instead raises the question on whether the deposits of secretory IgA were altered locally in the children with appendicitis. We propose that this should be further evaluated in future studies, by analyzing secretory IgA deposits histologically in the appendices and/or in stool samples. An association between increased secretory IgA in feces could theoretically potentially serve as a non-invasive biomarker for appendicitis.

### Strengths and limitations

To our knowledge, serum concentrations of IgA have never previously been investigated in a pediatric population with suspected appendicitis. The study was prospective, making the collection of additional data uniform, and all serum IgA analyses were performed by one single person (B.R.), at the time blinded to the final diagnoses of the study participants.

Among the limitations of the study is the fact that our IgA analyses were confined to serum IgA in blood samples, and that the production of secretory IgA in the appendices were not studied. Furthermore, the study was limited to a single center and the small sample size for the control group might make it more difficult to detect small to moderate differences in serum IgA, not the least due to the broad IQRs of the biomarker. Among the controls, the majority were diagnosed with “unspecified abdominal pain”, meaning that we cannot rule out other inflammatory conditions among these subjects. Since all hospitals in Skåne county use the same electronic health record system and all medical records were reviewed at least a couple of weeks after inclusion in order to have the finished reports from histopathological examinations, we are certain that no children in the non-appendicitis group were treated for appendicitis in the county during this time period.

Since the association between serum IgA and complicated appendicitis had not previously been studied in children an effect size could not be estimated, and therefore a power calculation was not carried out.

## Conclusion

In this first ever pediatric cohort study on associations between serum IgA and appendicitis, there were no significant associations between serum IgA and appendicitis or complicated appendicitis, neither with absolute concentrations nor after stratification into the categories normal, high, and low serum IgA according to age-dependent reference intervals. However, the modestly sized study cohort might make small or moderate differences hard to detect. Furthermore, the study is confined to analyzing IgA concentrations in serum. Therefore, our results do not rule out local IgA responses in the inflamed appendix, which should be the target of future research with measuring of secretory IgA in for example feces or in the appendix mucosa.

## Data Availability

Data supporting the results presented in this article will be made available by the corresponding author upon reasonable request.
